# Using virtual 3D-models in surgical planning: workflow of an immersive virtual reality application in liver surgery

**DOI:** 10.1007/s00423-021-02127-7

**Published:** 2021-03-12

**Authors:** Christian Boedecker, Florentine Huettl, Patrick Saalfeld, Markus Paschold, Werner Kneist, Janine Baumgart, Bernhard Preim, Christian Hansen, Hauke Lang, Tobias Huber

**Affiliations:** 1 Department of General-, Visceral- and Transplantation Surgery, University Medical Center Mainz, Langenbeckstr. 1, 55131 Mainz, Germany; 2 Department of Simulation and Graphics, Faculty of Computer Science, University of Magdeburg, Magdeburg, Germany

**Keywords:** Liver surgery, 3D reconstruction, Virtual reality

## Abstract

**Purpose:**

Three-dimensional (3D) surgical planning is widely accepted in liver surgery. Currently, the 3D reconstructions are usually presented as 3D PDF data on regular monitors. 3D-printed liver models are sometimes used for education and planning.

**Methods:**

We developed an immersive virtual reality (VR) application that enables the presentation of preoperative 3D models. The 3D reconstructions are exported as STL files and easily imported into the application, which creates the virtual model automatically. The presentation is possible in “OpenVR”-ready VR headsets. To interact with the 3D liver model, VR controllers are used. Scaling is possible, as well as changing the opacity from invisible over transparent to fully opaque. In addition, the surgeon can draw potential resection lines on the surface of the liver. All these functions can be used in a single or multi-user mode.

**Results:**

Five highly experienced HPB surgeons of our department evaluated the VR application after using it for the very first time and considered it helpful according to the “System Usability Scale” (SUS) with a score of 76.6%. Especially with the subitem “necessary learning effort,” it was shown that the application is easy to use.

**Conclusion:**

We introduce an immersive, interactive presentation of medical volume data for preoperative 3D liver surgery planning. The application is easy to use and may have advantages over 3D PDF and 3D print in preoperative liver surgery planning. Prospective trials are needed to evaluate the optimal presentation mode of 3D liver models.

**Supplementary Information:**

The online version contains supplementary material available at 10.1007/s00423-021-02127-7.

## Background

In hepatobiliary surgery, three-dimensional (3D) models of the liver with its vascular structures are reported to be helpful in preoperative planning by suggesting a shorter operation time, a reduction of the resection scope, or the amount of bleeding [1–3]. To date, these 3D reconstructions are mostly presented as 3D PDFs and only rarely as 3D-printed models. Some publications describe the presentation in preoperative planning by means of a 3D-printed model [4, 5] that gives a good impression of tumor location especially in multifocal tumor burden and when vascular invasion with the necessity of reconstruction is suspected [6]. The potential drawback of 3D prints is a time delay for the printing process and higher costs [7]. Viewing the model on a regular screen as a 3D PDF has the limitation of depending on the viewer’s mental ability to transfer images into 3D structures, but enables editing functions of the model like the change of the transparency of the different structures [8, 9]. With the technical progress and increasing affordable availability of virtual reality (VR) devices, medical applications have been introduced using VR as well. Thus, VR also represents an interesting new alternative for the presentation of these preoperative 3D models in medicine [10]. With this article, we introduce an immersive VR tool for interactive preoperative hepatobiliary surgery planning.

## Methods and workflow

To obtain a valid 3D visualization of the liver for surgical planning, a CT or MRI scan with contrasted hepatic artery and portal and hepatic veins must be available. The reconstruction can either be performed by an external provider or locally with (semi-)automatic segmentation software. In our department, semi-automatic, server-based software (Synapse 3D, FUJIFILM Europe GmbH, Düsseldorf) is utilized by trained members of our surgical team [11]. DICOM (Digital Imaging and Communications in Medicine) data can easily be transferred from the radiologic PACS System (Sectra AB, Linköping, Sweden) to synapse 3D. Transfer and reconstruction take about 2 h as previously described [11]. After completion of the reconstruction, the visualization is exported as stereolithography (STL) files, which are the standardized file format for 3D printing. The different structures (parenchyma, tumor, hepatic veins, portal vein, hepatic artery) are exported as separate files (Fig. 2).

Our application, which was developed using the game engine Unity (version 2019.2.14f1, Unity Technologies, San Francisco, CA, USA) enables an easy upload of these STL files (via drag and drop) and automatically generates the VR 3D model. The duration of the data transfer of the STL files and the calculation of the VR model takes no longer than 1 min. The application provides native support for all OpenVR compatible VR headsets, including the HTC Vive Pro (HTC Corporation, Taoyuan City 330, Taiwan), which is used in the current setup. The surgeon can view and edit the 3D model while wearing the VR headsets. To interact with the 3D liver model, both HTC Vive controllers can be used. Moving and rotating of the model are realized via a virtual “laser pointer.” By pointing towards the model and pressing a specific button, the model is attached to the intersection point and can be moved and rotated. To provide finer control over rotation, it is possible to use the touchpad of each controller: By pressing up/down or left/right, the model is rotated around the vertical or horizontal axis. Additionally, the model can be scaled by pressing and holding dedicated buttons on both controllers simultaneously; moving the controllers apart increases the model size and vice versa.

**Fig. 1:**
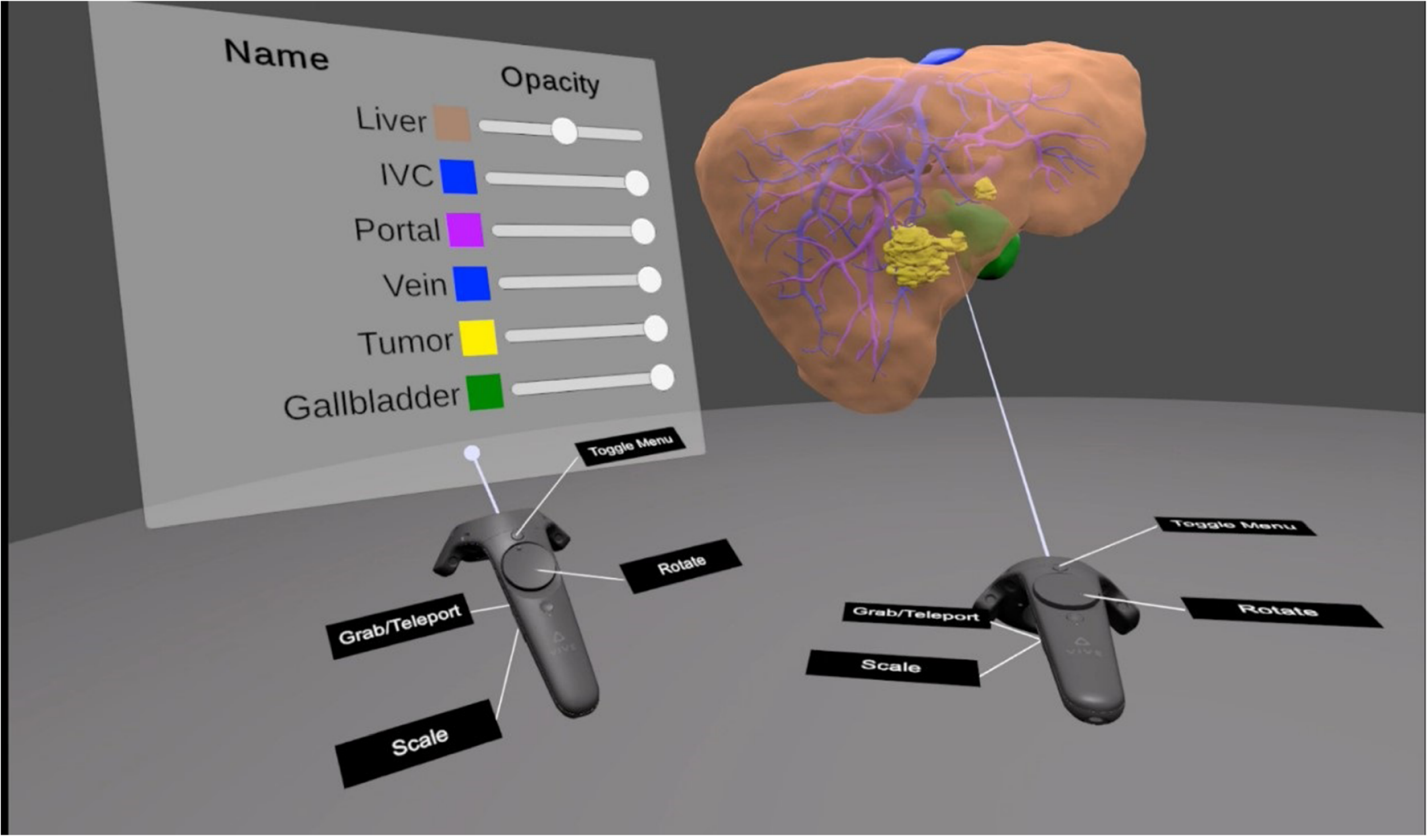
Virtual reality application for the preoperative demonstration of 3D liver models

A menu that can be shown and hidden as required by pressing a certain button gives an overview of all available structures. By interacting with a slider, the opacity can be changed from invisible over semi-transparent to fully opaque. In addition, surgeons can draw potential resection lines on the liver surface. All these functions can be used in a single or multi-user mode, which enables an interactive preoperative planning and discussion by the complete surgical team (Fig. 1, Supplementary Video 1)

ESM 1(MP4 195833 kb).

### Brief clinical evaluation

Aside from the incremental feedback from the clinical development team, we asked five highly experienced HPB surgeons of our department to evaluate the application after using it for the first time. Neither of  the five surgeons had any experience with VR technology before. The evaluation was carried out using the established “System Usability Scale” (SUS) [12]. Ten items on the system’s practicability and complexity as well as the user’s confidence to use and re-use it are rated on 5-point Likert scales. The evaluation using the Likert scale can be converted to a value of 0–100 by a fixed formula and would represent the best possible applicability at the maximum value of 100% (0–50%: not acceptable, 51–67%: poor, 68%: OK; 69–80%: good, 81–100%: excellent). The five evaluating surgeons from our department reached an average value of 76.6 %, which classified the application as rather easy to use, with 4 out of 5 surgeons giving a good to very good rating (80–95 %). One surgeon was not convinced by the application in its current form for daily clinical use, which resulted in a rating of 45%. Nevertheless, the participant emphasized the future potential. All 5 surgeons rated the learning effort to use the system as low (0.2 on the Likert scale 0–4), which positively highlights the system’s ease of use. Additionally, we discussed the potential use of the application and the competing demonstration modalities (PDF, printed model, and AR) (Table 1).

**Table 1:** Advantages and disadvantages of different presentation modalities of 3D liver reconstructions

Modality	Advantages	Disadvantages	Potential clinical application
3D PDF	- Low costs	- Missing actual 3-dimensionality (possible with additional hardware only)	- Tumorboard (on big monitor, 1 person interaction)
	- Show and hide structures	- Interaction limited (e.g., scaling, cutting)	- Intraoperative use (on monitor only)
			- Training
		- Multi-user interaction not supported	- Student teaching
			- Operation planning
3D print	- Natural model rotation and grasping	- High production and material costs	- Intraoperative use (sterile covering possible)
			- Patient information
	- Haptic interaction	- Time delay due to processing	- Student education
	- Original size	- Interaction limited (e.g., scaling, cutting)	- Operation planning
3D VR	- Show and hide structures	- Equipment needs to be available	- Advanced Surgical education
	- Advanced interaction such as scaling possible	- No haptic interaction (possible with additional hardware)	- Student education
	- Natural interaction possible, e.g., two-hand-interaction or grasping	- Technical knowledge necessary	- Virtual individual Tumorboard (if hardware available)
	- Multi-user interaction possible, also over distance	- Potentially cybersickness	- Operation planning
			- Simulation of surgery (perspective)
	- Simulation of surgery with resection mode		
3D AR	- Show and hide structures	- Equipment needs to be available - No haptic interaction (may be possible with additional hardware) - Technical knowledge necessary	- Intraoperative use (currently spectating, perspective with navigation) - Tumorboard (perspective, if enough glasses available) - Operation planning (interaction currently limited)
	- Advanced interaction such as scaling possible		
	- Natural interaction possible, e.g., two-hand-interaction or grasping (if possible to program)		
	- Multi-user interaction possible, also over distance		

*3D PDF*, reconstruction displayed as an interactive PDF file on a regular screen; *3D print*, full-size 3D-printed model of a liver reconstruction; *3D VR*, demonstration of the 3D liver model using virtual reality (VR); *3D AR*, demonstration of 3D models using augmented reality (AR)

The demonstration of the system to the HPB experts was independent of a specific surgical procedure. Thus, we used cases that were available in our collection of 3D reconstructions that have been printed for surgical planning [6]. Thus, intraoperative feedback regarding the preoperative visualization in immersive VR from the surgeons was not collected and will be part of future investigations in a clinical trial.

## Discussion

**Fig. 2:**
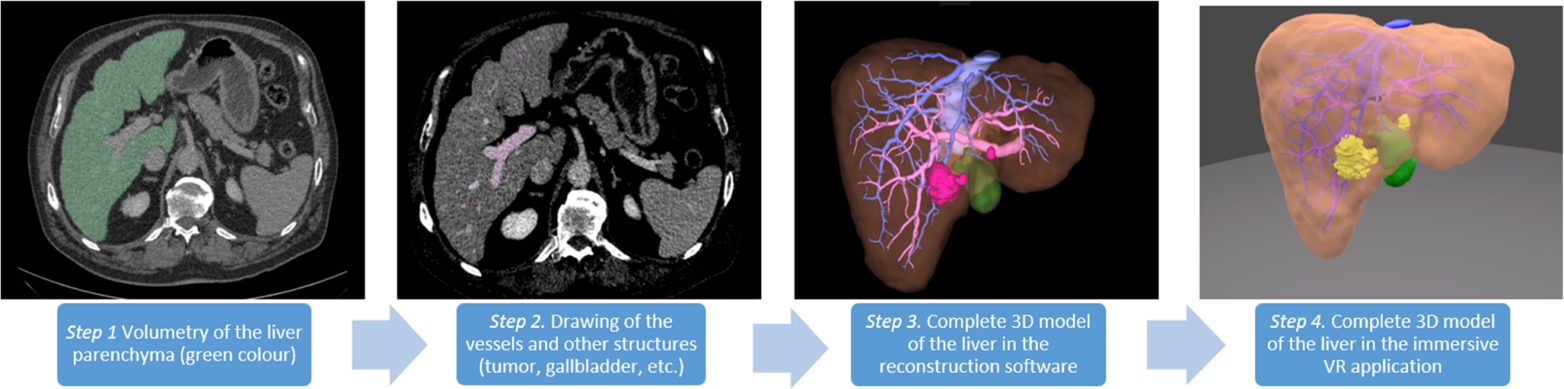
Workflow of the 3D model creation

Until today, there is little literature about the application of VR as a representation modality of 3D models in liver surgery. Most publications describe the representation of such models in preoperative planning by means of 3D PDF or 3D prints [4, 8]. The term virtual resection planning is often misleading and usually refers to the ability to draw resection lines on a reconstructed liver with volume calculation. However, these applications are currently presented on 2D monitors. We have developed an immersive application that demonstrates 3D models in virtual reality using VR headsets and furthermore enables interaction with the model (Supplementary Video 1). An essential requirement for a VR application as a presentation modality for preoperative surgical planning is the correct presentation of the original data. This is ensured by this application (Fig. 2). In addition, this technology avoids the disadvantages of viewing the models on a 2D screen, and at the same time, it enables almost all advantages of 3D printing, especially moving and grasping the models. In VR, these movements can be carried out with controllers. The drawback of surface reflection, which can arise from the surface of the 3D print model at certain angles, is also avoided in VR. Furthermore, various interactions can be used to “show or hide” overlaps of pathologies and vessels of the liver in the VR application for better understanding. This is not possible with a 3D print. However, the lacking haptic interaction with the 3D model with the surgeon’s own hands but with the controllers can be a limitation of the VR application and will be a part of future research by combining 3D prints and VR [13] or with specialized VR gloves. One advantage of imaging in a 3D pdf format and viewing on a 2D screen—besides the broad availability and low costs—is currently the possibility to enable volume calculation, provided that a volume segmentation of the liver was initially performed and specially exported. This is not yet included in this application but part of current investigations. However, for an accurate and on-demand volume calculation directly in the immersive VR application, proper data format is DICOM instead of the currently used STL files.

The intraoperative use of the 3D visualization of preoperative image data is a field that has been widely investigated. Recent developments in augmented reality (AR) technologies enable the spectating of 3D reconstructions in a sterile field using speech or gesture commands [14, 15]. The live alignment of the preoperative data in the operating room during liver surgery with the currently available AR technology is lacking accuracy but will hopefully be possible with technological advances. The intraoperative use of this VR application is conceivable, but at the moment certainly limited due to the loss of sight of the actual surgical field. Another limitation is the initial costs for the VR equipment as well as the increased technical effort due to the different computer programs and their handling. The advantages of the more established 3D presentation modalities may be that a 3D-printed model can enable a more plastic preoperative patient information about their upcoming surgery [5]. A 3D PDF may be suitable when the presentation on a screen covers the need e.g. of a tumor board. However, virtual meetings and the presentation of 3D data in immersive VR are already possible and will probably gain wider acceptance and use [16]. Also, medical education and specialized HPB apprentices may profit from immersive virtual reality applications due to the interactive use and the possibility to interact with multiple participants and over distances. This is in concordance with recent literature that evaluated a similar VR application to be suited mostly for student and resident training [17]. To date, this is the main development goal of the presented application. However, all these possibilities have to be put into perspective since technological advances will make VR and AR applications more available and more affordable for a broad field of users. The integration of patient data into VR applications needs to be evaluated regarding the connection to hospital information systems (HIS) with the necessary data security ensured. Table 1 gives an overview of the advantages and disadvantages of the mentioned technologies as feedback from the evaluating HPB specialists. However, trials are needed to evaluate which presentation modality suits the different areas of application in surgery, e.g., preoperative planning or education.

In conclusion, we present an immersive VR application for preoperative 3D liver surgical planning. The application is easy to use and may have advantages over 3D PDF and 3D print in preoperative liver surgery planning. Prospective trials are needed to evaluate the optimal presentation mode of 3D liver reconstructions.
